# Health-Related Quality-of-Life among Pregnant Women after First, Second, and Multiple Cesarean Sections in the Perinatal Period: A Short-Term Longitudinal Study

**DOI:** 10.3390/ijerph192416747

**Published:** 2022-12-13

**Authors:** Michalina Ilska, Anna Kołodziej-Zaleska, Ewa Banaś-Fiebrich, Anna Brandt-Salmeri, Ewa Janowska-Tyc, Anna Łyszczarz, Justina Rzewiczok, Bogusława Piela, Wojciech Cnota

**Affiliations:** 1 Institute of Psychology, University of Silesia in Katowice, Grażyńskiego Street 53, 40-126 Katowice, Poland; 2 Clinical Department of Perinatology, Gynaecology and Obstetrics in Ruda Ślaska, Medical University of Silesia, W. Lipa Street 2, 41-703 Ruda Śląska, Poland; 3 Faculty of Health Sciences in Katowice, Medical University of Silesia, 41-703 Ruda Śląska, Poland

**Keywords:** health-related quality-of-life, multiple cesarean section, health problems, pregnancy

## Abstract

The primary objective of this study was to compare assessments of health-related quality-of-life (HRQoL) in women who had a medical qualification for cesarean section (CS), depending on the number of CSs in their medical history. A short longitudinal study was conducted among 115 women on the day before a planned cesarean section (CS)-T1, and on the third day after CS-T2. They were divided into three groups. G1: no CS (*n* = 17); G2: one CS (*n* = 34); G3: two or more CSs (*n*  =  64). Participants completed a set of questionnaires concerning sociodemographic aspects and psychological outcomes: the HRQoL questionnaire (EQ-5D-3L). A chi-square test, McNemar’s test, and repeated measures ANOVA were used to compare the three groups in T1 and T2. Regardless of the number of CSs, before a CS, women mainly experience health problems with pain and anxiety/depression, and after a CS, mostly problems with pain, usual activity, and mobility. All participants experienced an increase in the amount of health problems with mobility and pain after a CS. Women who have had two or more CSs also had problems with self-care and usual activities. Women who have had one or two CSs experienced a decrease in the general assessment of the HRQoL, which is not observed in the group of women with multiple CSs. However, the HRQoL of women in the group with multiple CSs was lower before the CS than in the other groups. The results indicated the significance of the number of CSs, not only in postoperative, but also in preoperative HRQoL.

## 1. Introduction

Cesarean section (CS) is a surgical procedure by which a newborn is delivered when a vaginal birth is contraindicated or unachievable. The most common indications for this procedure are: failed progress of labor, fetal distress, fetal malpresentation, and a history of cesarean delivery [1]. The World Health Organization (WHO) reports a remarkable increase in CS rates, even though the average CS worldwide rate is supposed to be 10–15% at the highest [2]. In Poland, this percentage is much higher, at approximately 43%, being one of the highest in Europe [1,3].

Apart from the fact that it is often, undeniably, a necessary procedure, CS is associated with increased maternal and neonatal morbidity, including postpartum infection, hemorrhage, venous thromboembolic disease, and postpartum depression [4,5,6]. Cesarean delivery can also lead to adverse consequences in subsequent pregnancies, such as placenta accreta spectrum (PAS) [7,8] or uterine scar rupture [1]. According to the literature, women who have CSs have a lower quality-of-life when compared to those who have a vaginal delivery [6,9], and are exposed to a risk of persistent pain [10].

As the CS rate increases, there are more and more patients who have undergone multiple cesarean sections. These patients are proven to be at a higher risk of hysterectomy, blood transfusions, intra-abdominal adhesions, surgical injuries of surrounding organs, wound infection, placenta previa, and PAS [8,11,12]. General morbidity rises constantly with each successive CS [12]. Recovery after subsequent CSs can also be longer and more painful [1]. This can have a significant impact on women’s quality-of-life during pregnancy and after the next CS.

There are still few studies focusing on postpartum quality-of-life after CS and during the next pregnancy. Moreover, there is a lack of research on quality-of-life after multiple CSs. Few studies focus on the assessment of quality-of-life in women after childbirth comparing the type of birth: CS versus vaginal delivery (VD) [13,14,15]. Some results have shown that women after cesarean section have a lower quality-of-life both in the puerperium and up to a year after the CS [14,15]. Long-term differences in quality-of-life between women after a CS and after VD have also been highlighted by other researchers [16,17].

Undoubtedly, the convalescence of women after successive cesarean sections is longer and more painful [1], and the pain and fatigue they experience may negatively affect their quality-of-life [10]. Women also experience more depressive and pain symptoms after a cesarean section [6]. This might significantly affect their quality-of-life during their next pregnancy and the postpartum period, as well as their experiences of parenthood and future family planning decisions.

This research aims to compare assessments of health-related quality-of-life in women who had a medical qualification for a CS, depending on the number of CSs in their medical history.

## 2. Materials and Methods

The study was conducted between May 2021 and September 2022 at the Clinical Department of Perinatology, Gynecology, and Obstetrics in Ruda Śląska, which is a 3rd level reference department. We compared 115 women with medical inclusion for cesarean section (CS). We divided them into 3 groups depending on the number of CSs declared during the obstetric interview: Group 1: no CS (*n* = 17); Group 2: one CS (*n* = 34); Group 3: two or more CSs (*n*  =  64—multiple group). Data on the obstetrician, surgical, and anesthetic procedure were retrieved from the medical records. Verbal and written informed consent to participate in the research was obtained from all participants. All patients gave their consent for their data to be used for scientific purposes. Approval was obtained from the Ethics Committee of the University of Silesia in Katowice, Poland (no. KEUS.121/04.2021).

### 2.1. Health-Related Quality-of-Life Assessment

The Polish version of the EQ-5D-3L questionnaire was used. The EQ-5D-3L is a generic health quality-of-life (HRQoL) instrument [18]. The questionnaire consists of two parts. The descriptive system assesses self-reported health in five dimensions: mobility, self-care, usual activities, pain/discomfort, and anxiety/depression, rated as “no problems”, “some problems”, or “extreme problems”. Patients also rated their health on the EQ visual analog scale (EQ-VAS) from 100 mm “best imaginable health state” to 0 mm “worst imaginable health state”.

### 2.2. Obstetric Outcomes

Data on the course of the current pregnancy (miscarriages, week of pregnancy, number of previous births, placenta problems: yes/no) and the health of the respondents (BMI, occurrence of chronic diseases, stimulants) were collected from medical records and using our own questionnaire.

### 2.3. Operation and Anesthetic Outcomes

Data on the type of anesthesia, type of incision, course of surgery (hysterectomy: yes/no), presence of adhesions, blood loss, and length of surgery were collected from the medical records.

### 2.4. Inclusion and Exclusion Criteria

**Figure 1:**
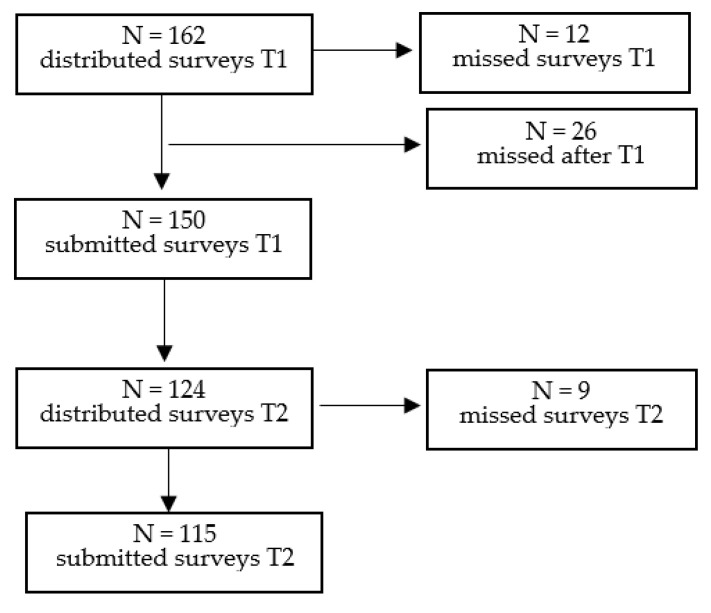
Process of study selection.

Pregnant women, aged 18–49, with single pregnancies, scheduled for an elective CS in the third trimester, and understanding the Polish language were considered eligible for inclusion. Ongoing treatment for chronic pain or psychiatric disorder, or a history of illegal drug abuse, constituted exclusion criteria. Furthermore, cases with missing surveys had to be excluded (*n* = 47; cf. Figure 1).

### 2.5. Statistical Analysis

The collected data were analyzed using SPSS statistical software version 26 and JASP 0.16.0.0. The sample size calculation was based on the sample size for previous research on quality-of-life after the cesarean section [19]. Descriptive statistics were used to describe the basic characteristics of the participants. Data were reported as mean (SD) for continuous variables. Nominal data are presented as numbers and percentages. HRQoL was compared for women qualifying for a CS with a different number of CSs in their medical history on the day before the surgery (T1) and on day 3 after the surgery (T2). A chi-square test and McNemar’s test were used to analyze the categorical data. To describe the HRQoL, descriptive statistics, including the mean and standard deviation (SD), were used. The repeated measures ANOVA was used to evaluate the EQ-VAS scores time trend in the three groups. In all calculations, a level of *p* ≤ 0.05 was considered significant.

## 3. Results

In this study, we enrolled 115 pregnant women, eligible for a CS, who were divided into three groups depending on the number of CSs declared in the obstetric interview: Group 1: no CS (*n* = 17); Group 2: one CS (*n* = 34); Group 3: two or more CSs (*n*  =  64). The mean age of the women was 32.89 (4.61), with a range from 22 to 45 years old.

The differences between the groups were found to be statistically significant in terms of mean maternal age, relationship status, and work status. No statistically significant difference was observed between the groups with respect to financial status or education (see Table 1).

The differences between the groups were found to be statistically significant in terms of mean operation time and blood loss. In addition, the differences were also statistically significant for unplanned pregnancy, type of incision, and severe adhesion. No statistically significant difference was observed between the groups with respect to miscarriages, high-risk pregnancy, chronic medical condition, premature birth, length of hospital stay, prenatal diagnosis of placental abnormalities, type of anesthesia, or cesarean hysterectomy (see Table 2).

### 3.1. Health Problems

Because the reported level 3 problems were low, as suggested by the questionnaire guideline, we dichotomized the EQ-5D levels into “no problem” (level 1) and “problems” (level 2 or 3). In the entire sample in T1, pain/discomfort problems occurred most frequently (53%), followed by anxiety/depression (51.3%) and usual activities (38.3%). Problems with self-care (12.2%) and mobility (22.6%) were least frequent. In all five dimensions, the number of reported health problems before and after the CS in the study groups was at a similar level.

In the entire sample in T2, pain/discomfort problems occurred most frequently (91.3%), followed by usual activities (60.0%) and mobility (54.8%). Problems with self-care (36.5%) and anxiety/depression (31.3%) were least frequent. In all five dimensions, the number of reported health problems before and after CS in the study groups was at a similar level, except pain/discomfort problems (*χ*^2^ = 8.72; *p* = 0.01), which was the most frequent in groups 1 and 2 in T2 (G1 = 100%; G2 = 100%; G3 = 84.4%).

**Figure 2:**
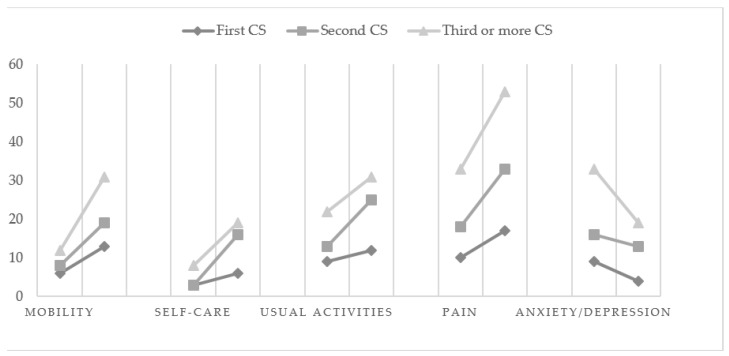
Any problems reported by the EQ-5D-3L dimensions in T1 and T2 (*n* = 115).

The analysis with McNemar’s test revealed whether the number of health problems in each dimension changes with time (T1 vs. T2) in the studied groups. The number of respondents reporting any problems in each EQ-5D-3L dimension in T1 and T2 is shown by group in Figure 2. The conducted analysis showed that, for mobility, more problems were experienced by women in T2, and the change was statistically significant in all groups (G1: *χ*^2^ = 5.14; *p* = 0.02; G2: *χ*^2^ = 7.69; *p* = 0.06; G3: *χ*^2^ = 12.00; *p* = 0.001). For self-care, more problems were reported by women in T2, but there was only a significant change in the G2 and G3 groups (G1: *χ*^2^ = 0.80; *p* = 0.37; G2: *χ*^2^ = 11.07; *p* = 0.001; G3: *χ*^2^ = 4.76; *p* = 0.02). For usual activities, more problems were also experienced by women in T2, and a significant change was observed in the G2 and G3 groups (G1: *χ*^2^ = 0.80; *p* = 0.37; G2: *χ*^2^ = 8.64; *p* = 0.003; G3: *χ*^2^ = 3.76; *p* = 0.04). For pain/discomfort, more problems were observed in T2, and a significant change was observed in all groups (G1: *χ*^2^ = 5.14; *p* = 0.02; G2: *χ*^2^ = 13.06 *p* = 0.001; G3: *χ*^2^ = 12.89; *p* = 0.001). For anxiety/depression, more problems were observed in T1, but a significant change was observed only in the G3 group (G1: *χ*^2^ = 2.28; *p* = 0.13; G2: *χ*^2^ = 0.44; *p* = 0.50; G3: *χ*^2^ = 7.04; *p* = 0.008).

### 3.2. EQ-VAS Scores

**Figure 3:**
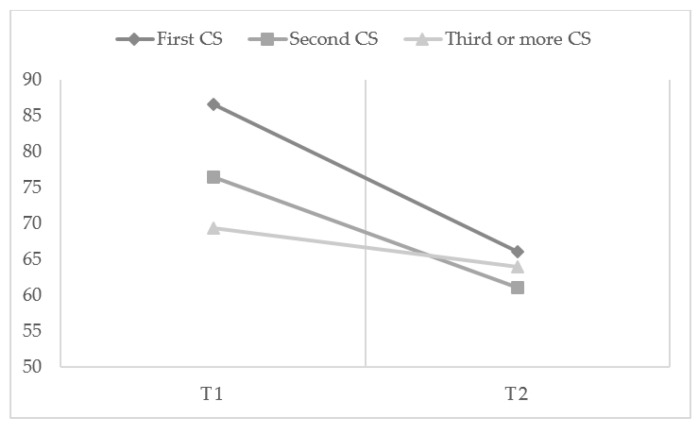
Time trend of EQ-VAS scores in cesarean section (CS) groups.

A repeated measurement analysis was carried out to check the time trend (T1 vs. T2) for EQ-VAS scores between the groups. The between-groups test in the repeated measurement analysis (Figure 3) showed that the effect of “group” was not significant (*p* = 0.102). The within-subject test indicated a significant time effect, demonstrating that the groups did change over time (*p* < 0.001). Two groups—Group 1 (*p* = 0.003) and Group 2 (*p* = 0.001)—saw a significant decrease in EQ-VAS scores three days post-CS. The effect of interaction between time and group was also found to be significant (*p* = 0.017), suggesting that the effect on the groups differed over time. All groups had a different EQ-VAS score initially, which decreased over time with different slopes, leading to different scores at three days post-CS.

Because the effect of interaction between time and group was significant, the EQ-VAS scores in the three groups were compared at each time point. No difference was identified in the mean EQ-VAS score at baseline between the G1 and G2 groups (86.58 ± 8.89 vs. 76.52 ± 19.48 in the G1 group and G2 group, respectively, *p* = 0.532). The EQ-VAS score was higher at baseline CS in the G1 group than in the G3 group (G1 = 86.58 ± 8.89 vs. G3 = 70.52 ± 19.22, *p* = 0.019). On the third day post-CS, the mean EQ-VAS scores were 66.00 (13.65) in G1, 61.02 (16.42) in G2, and 63.92 (23.18) in G3, which was not found to be statistically different (see Figure 3).

## 4. Discussion

The results of the presented study indicate differences in the assessment of the health-related quality-of-life (HRQoL) between the groups of women who had a medical qualification for cesarean sections (CS), depending on the number of CSs in their medical history, and depending on the measured time point (T1—before cesarean section, and T2—3 days after surgery). The statistical analysis showed that women who have had a first and second CS rated their health as worse, using the EQ visual analog scale (EQ-VAS), compared with their assessments before the CS. The women in these groups experienced a decrease in general HRQoL. The decrease in the assessment of the general HRQoL was not significant in the group of women who had had three or more cesarean sections. The experience of a cesarean section procedure at least twice in the multiple CS group could also have given some kind of “psychological preparation” for what the procedure entails, with postprocedure discomfort therefore being less of a shock. Some women, especially those with no history of CS, might consider a cesarean section delivery to be less valuable, so they might feel disappointed at losing their opportunity for a natural birth. These sentiments might worsen their perception of their own health and bodily capabilities, decreasing their HRQoL as a result. However, this is only a hypothetical explanation for the lower HRQoL after surgery among women following their first CS. The other explanation may be that the assessment of their health before the cesarean section was already low, and significantly lower than for women who have not yet experienced a CS. Our study showed the significance of the number of CSs, not in the postoperative general HRQoL, but in the preoperative HRQoL. The perceived HRQoL level after cesarean section did not differ between the study groups; however, before the CS, it was lower in the multiple CS group than in the group of women with no history of CS. It is difficult to compare the obtained results of the research, as most of the analyses concern comparisons between the type of delivery and not the number of CSs. The available research indicates a worse quality-of-life after a CS compared to vaginal delivery; however, these studies do not take into account the number of CSs [13,16,17,19].

Further analysis in this area is necessary. In the case of studies on multiple cesarean sections, it would be particularly important to compare a large group of women with a different number of cesarean sections, as well as taking into account longer time periods after the CS. Women’s health-related quality-of-life might also be affected by the type of procedure performed (for example, the Charité cesarean birth or “family-centered cesarean”), as well as possible surgical complications. These aspects have also been highlighted in other studies [4,11,20,21].

Extensive analysis of health-related problems in the studied group of women shows that pain/discomfort and anxiety/depression were the main complaints reported by over 50% of women before delivery, regardless of the number of CSs in their history. Problems with usual activity, mobility, and self-care before a CS were reported by a smaller percentage of women. However, after a CS delivery, regardless of the number of CSs before, pain/discomfort was reported by more than 90% of women. Problems with usual activities (60%), mobility (over 50%), and self-care (33%) were also more frequent. Fewer women complained of mood problems such as anxiety or depression—less than 33%. Previous research shows that experiencing pain/discomfort problems after a CS can last up to a year [16].

Medical complications increase the risk of many health problems and could be relevant to the lower assessment of HRQoL in the group of women with multiple CSs. Previous studies have found that multiple CSs was associated with an increased risk of morbidity [22], placenta previa, placenta accrete, uterine dehiscence or rupture, postpartum hemorrhage, blood transfusion, bladder injury, longer operative time and hospital stay, and adhesion formation [1,8,11,23]. Our study shows that every subsequent CS is associated with longer operative time, higher blood loss, adhesion occurrence, and more frequent vertical incisions in women who have had multiple CSs.

Comparing whether and how the number of health problems in each dimension changes with time, before and after a CS, we can see that in all dimensions of health problems, women experienced an increase in the number of problems after a CS. All study groups experienced an increase in problems with pain and mobility after a CS. In addition, women who have had two or more cesarean sections experienced more problems with self-care and usual activities after a CS. An increase in problems with self-care and usual activities was not experienced by women after their first CS. Interestingly, in the case of anxiety/depression, women experienced a decrease in the number of problems after multiple CSs. Perhaps the emotional component of the predelivery problems in the case of three or more CSs was related to the fear of the surgery itself, burdened with a high risk of complications for the mother and child. Further analyses are advisable. The literature includes a review of studies pointing to the role of anxiety and depression in various types of labor [6,24], but the studies concern only elective CSs on maternal request and women who delivered vaginally, or the effectiveness of interventions aimed at reducing the fear of CSs. There is a lack of research investigating the HRQoL of women who have had multiple CSs, although this issue seems to be important, especially due to the medical documentation of the basis of potential health problems for both mother and child and their medical and psychological repercussions [25,26,27,28].

### 4.1. Implications for Practice and/or Policy

The assessment of quality-of-life in women who have had multiple CSs shows potential aspects of women’s health that physicians should focus on during the perioperative period. The use of appropriate medical procedures and treatment appears to be a key factor in perinatal care to improve maternal health.

The presented analysis indicates the need to raise awareness of the long-term risks of CSs, especially in those women who would like to have three or more children.

Considering the risk of medical complications and health-related problems, it is crucial to choose the method of delivery carefully and revise cesarean section indications. Undertaking further research on the assessment of the quality of life of pregnant and postpartum women who have had multiple CSs would allow for the broadening of knowledge in this area and the creation of adequate intervention plans and routines for medical personnel, as well as educational programs for pregnant women and their partners. It is important to verify the indications for the first and subsequent cesarean sections and to increase women’s the awareness about the possible effects of subsequent cesarean sections on their health and quality-of-life.

### 4.2. Limitations and Strengths

Several limitations of the present research should be noted. Firstly, our sample was relatively small and consisted of pregnant women, most of whom were married or in a close relationship, highly educated, and working full-time. The sample was, thus, rather sociodemographically homogeneous, and caution is needed in generalizing these findings to the entire population of pregnant women. In future investigations, it will be necessary to maximize diversity among participants. Secondly, the future use of tools for measuring the severity of specific problems on an interval scale would allow the use of advanced statistical analysis to assess changes in the studied phenomenon. It is also important to extend the examination time by further time points to better understand the trajectory of changes in the quality-of-life of women following a cesarean section. Despite these limitations, we believe that this study enriches our knowledge on the changes in health-related quality-of-life for women in the perinatal period.

## 5. Conclusions

Health-related quality-of-life depends on the number of past cesarean sections.

Early puerperium is associated with a significant decrease in quality-of-life and the number of problems experienced by women following a cesarean section.

Moreover, multiple cesarean deliveries are associated with more difficult and complicated surgery compared with a first planned cesarean delivery. Women’s health problems experienced in the pre- and postoperative period are variable and require adequate perinatal care.

It is important to verify the indications for the first and subsequent cesarean sections and to increase women’s the awareness about the possible effects of subsequent cesarean sections on their quality-of-life.

## Figures and Tables

**Table 1:** Sociodemographic characteristics of the participants (*n* = 115).

	Group 1 *n* (%)	Group 2 *n* (%)	Group 3 *n* (%)	Test; *p*
Age (years) M (SD)	30.64 (3.10) ^a^	32.14 (4.51) ^a^	34.48 (4.80) ^b^	*T* = 12.47
				*p* = 0.002
Relationship status				*χ*^2^ = 14.12 *p* = 0.007
Married	14 (82.4)	32 (94.1)	44 (68.8)	
In a relationship	1 (5.9)	2 (5.9)	18 (28.1)	
None	2 (11.8) ^a^	0 (0.0)	2 (3.1)	
Financial status				*χ*^2^ = 6.57 *p* = 0.160
Below average	1 (5.9)	4 (11.8)	4 (6.8)	
Average	11 (64.7)	28 (82.4)	40 (67.8)	
Above average	5 (29.4)	2 (5.9)	15 (25.4)	
Education				*χ*^2^ = 11.68 *p* = 0.06
Primary school	0 (0.0)	0 (0.0)	9 (14.5)	
Vocational	0 (0.0)	2 (5.9)	7 (11.3)	
High school	4 (25.0)	11 (32.4)	14 (22.6)	
Postgrad	12 (75.0)	21 (61.8)	32 (51.6)	
Work				*χ*^2^ = 20.14 *p <* 0.001
Yes, full-time job	15 (88.2)	30 (88.2)	31 (49.2)	
Yes, part-time job	1 (5.9)	2 (5.9)	8 (12.5)	
No	1 (5.9)	2 (5.9)	24 (37.5)	

Note. Group 1: no CS; Group 2: one CS; Group 3: two or more CSs. Means with different superscripts are significantly different at *p* < 0.05 in a post hoc test.

**Table 2:** Obstetric, anesthetic, and operational characteristics of all groups (*n* = 115).

	Group 1 *n* (%)	Group 2 *n* (%)	Group 3 *n* (%)	Test; *p*
Obstetric Characteristic				
BMI, M (SD)	28.84 (6.12)	29.78 (5.91)	28.65 (28.65)	*T* = 0.48 *p* = 0.786
Gestational age at delivery (weeks)				
M (SD)	37.82 (1.50)	37.61 (1.92)	37.37 (1.75)	*T* = 2.01 *p* = 0.366
Unplanned pregnancy				*χ*^2^ = 12.56 *p* = 0.002
No	10 (58.8)	30 (88.2)	33 (51.6)	
Yes	7 (41.2)	4 (11.8)	30 (46.9)	
Miscarriages				*χ*^2^ = 1.47 *p* = 0.372
Yes	4 (23.5)	10 (29.4)	24 (37.5)	
No	13 (76.5)	24 (70.5)	40 (62.5)	
High-risk pregnancy				*χ*^2^ = 6.34 *p* = 0.175
Yes	6 (35.3)	13 (38.2)	37 (57.8)	
No	7 (41.2)	17 (50.0)	19 (29.7)	
Unsure	4 (23.5)	4 (11.8)	8 (12.5)	
Chronic medical conditions				*χ*^2^ = 1.74 *p* = 0.580
Yes	7 (41.2)	8 (23.5)	18 (28.1)	
No	10 (58.8)	26 (76.5)	46 (71.9)	
Prenatal diagnosis of placental abnormalities				*χ*^2^ = 0.13 *p* = 0.831
Yes	2 (11.8)	4 (11.8)	9 (14.1)	
No	15 (88.2)	30 (88.2)	55 (85.9)	
Hospital stay (days) M (SD)	9.82 (13.03)	8.17 (9.26)	10.51 (13.09)	*T* = 3.44 *p* = 0.179
Premature birth				*χ*^2^ = 0.61 *p* = 0.736
Yes	4 (23.5)	5 (14.7)	12 (18.8)	
No	13 (76.5)	29 (85.3)	52 (81.3)	
Operation Characteristic				
Anesthesia				*χ*^2^ = 3.07 *p* = 0.215
General	0 (0.0)	4 (11.8)	10 (15.6)	
Regional (spinal/epidural)	17 (100)	30 (88.20	54 (84.4)	
Type of incision				*χ*^2^ = 10.81 *p* = 0.004
horizontal	13 (76.5)	20 (58.8)	23 (35.9)	
vertical	4 (23.5)	14 (41.2)	41 (64.1)	
Adhesions				*χ*^2^ = 9.82 *p* = 0.007
Yes	1 (2.5)	10 (29.4)	29 (45.3)	
No	16 (94.1)	24 (70.6)	35 (54.7)	
Blood loss M (SD)	270.58 (91.95)	485.29 (542.09)	440.62 (396.80)	*T* = 12.69 *p* = 0.002
Operation time M (SD)	40.29 (10.52) ^a^	56.82 (24.19) ^b^	56.82 (32.28) ^b^	*T* = 8.70 *p* = 0.013
Cesarean hysterectomy				*χ*^2^ = 2.06 *p* = 0.357
Yes	0 (0.0)	4 (11.8)	6 (9.4)	
No	17 (100)	30 (88.2)	58 (90.6)	

Note. Group 1: no CS; Group 2: one CS; Group 3: two or more CSs. Means with different superscripts are significantly different at *p* < 0.05 in a post hoc test.

## Data Availability

The datasets used and/or analyzed during the current study are available from the corresponding author upon request.

## References

[1] Wielgoś M., Bomba-Opoń D., Bręborowicz G.H., Czajkowski K., Dębski R., Leszczyńska-Gorzelak B., Oszukowski P., Radowicki S., Zimmer M. (2018). Recommendations of the Polish Society of Gynecologists and Obstetricians Regarding Caesarean Sections. Ginekol. Pol..

[2] Betran A.P., Torloni M.R., Zhang J.J., Gülmezoglu A.M. (2016). WHO Statement on Caesarean Section Rates. BJOG.

[3] Polish Ministry of Health Rządowy Program Kompleksowej Ochrony Zdrowia Prokreacyjnego w Polsce w 2021-2023 r.—Ministerstwo Zdrowia—Portal Gov.Pl. https://www.gov.pl/web/zdrowie/program-kompleksowej-ochrony-zdrowia-prokreacyjnego-w-polsce-w-2021-r.

[4] Mascarello K.C., Horta B.L., Silveira M.F. (2017). Maternal Complications and Cesarean Section without Indication: Systematic Review and Meta-Analysis. Rev. Saude Publica.

[5] Villar J., Carroli G., Zavaleta N., Donner A., Wojdyla D., Faundes A., Velazco A., Bataglia V., Langer A., Narváez A. (2007). Maternal and Neonatal Individual Risks and Benefits Associated with Caesarean Delivery: Multicentre Prospective Study. Br. Med. J..

[6] Ilska M., Banaś E., Gregor K., Brand-Salmerii A., Ilski A., Cnota W. (2020). Vaginal Delivery or Caesarean Section—Severity of Early Symptoms of Postpartum Depression and Assessment of Pain in Polish Women in the Early Puerperium. Midwifery.

[7] Creanga A.A., Bateman B.T., Butwick A.J., Raleigh L., Maeda A., Kuklina E., Callaghan W.M. (2015). Morbidity Associated with Cesarean Delivery in the United States: Is Placenta Accreta an Increasingly Important Contributor?. Am. J. Obs. Gynecol..

[8] Cnota W., Banas E., Dziechcinska-Poletek D., Janowska E., Jagielska A., Piela B., Czuba B. (2022). “The Killer Placenta”—A Threat to the Lives of Young Women Giving Birth by Cesarean Section. Ginekol. Pol..

[9] Kohler S., Sidney Annerstedt K., Diwan V., Lindholm L., Randive B., Vora K., de Costa A. (2018). Postpartum Quality of Life in Indian Women after Vaginal Birth and Cesarean Section: A Pilot Study Using the EQ-5D-5L Descriptive System. BMC Pregnancy Childbirth.

[10] Niklasson B., Georgsson Öhman S., Segerdahl M., Blanck A. (2015). Risk Factors for Persistent Pain and Its Influence on Maternal Wellbeing after Cesarean Section. Acta Obs. Gynecol. Scand..

[11] Marshall N.E., Fu R., Guise J.M. (2011). Impact of Multiple Cesarean Deliveries on Maternal Morbidity: A Systematic Review. Am. J. Obs. Gynecol..

[12] Grivell R.M., Dodd J.M. (2014). Short- and Long-Term Outcomes after Cesarean Section. Expert Rev. Obstet. Gynecol..

[13] Torkan B., Parsay S., Lamyian M., Kazemnejad A. (2009). Postnatal Quality of Life in Women after Normal Vaginal Delivery and Caesarean Section. BMC Pregnancy Childbirth.

[14] Bobek M., Humaj-Grysztar M., Matuszyk D., Put M. (2018). Quality of life assessment of the primiparas in early postpartum period depending on the mode of delivery. Polski Przegląd Nauk Zdrowiu.

[15] Van der Woude D.A.A., Pijnenborg J.M.A., de Vries J. (2015). Health Status and Quality of Life in Postpartum Women: A Systematic Review of Associated Factors. Eur. J. Obstet. Gynecol. Reprod. Biol..

[16] Petrou S., Kim S.W., McParland P., Boyle E.M. (2017). Mode of Delivery and Long-Term Health-Related Quality-of-Life Outcomes: A Prospective Population-Based Study. Birth.

[17] Sadat Z., Taebi M., Saberi F., Kalarhoudi M.A. (2013). The Relationship between Mode of Delivery and Postpartum Physical and Mental Health Related Quality of Life. Iran. J. Nurs. Midwifery Res..

[18] Golicki D., Jakubczyk M., Niewada M., Wrona W., Busschbach J.J.V. (2010). Valuation of EQ-5D Health States in Poland: First TTO-Based Social Value Set in Central and Eastern Europe. Value Health.

[19] Ghaffari S., Dehghanpisheh L., Tavakkoli F., Mahmoudi H. (2018). The Effect of Spinal versus General Anesthesia on Quality of Life in Women Undergoing Cesarean Delivery on Maternal Request. Cureus.

[20] Kram J.J.F., Montgomery M.O., Moreno A.C.P., Romdenne T.A., Forgie M.M. (2021). Family-Centered Cesarean Delivery: A Randomized Controlled Trial. Am. J. Obs. Gynecol. MFM.

[21] Radtke L., Dukatz R., Biele C., Paping A., Sameez K., Klapp C., Henrich W., Dückelmann A.M. (2022). Charité Caesarean Birth Improves Birth Experience in Planned and Unplanned Caesarean Sections While Maintaining Maternal and Neonatal Safety: A Prospective Cohort Study. Clin. Exp. Obs. Gynecol..

[22] Makoha F.W., Felimban H.M., Fathuddien M.A., Roomi F., Ghabra T. (2004). Multiple Cesarean Section Morbidity. Int. J. Gynecol. Obstet..

[23] Qublan H.S., Tahat Y. (2006). Multiple Cesarean Section. The Impact on Maternal and Fetal Outcome. Saudi. Med. J..

[24] Olieman R.M., Siemonsma F., Bartens M.A., Garthus-Niegel S., Scheele F., Honig A. (2017). The Effect of an Elective Cesarean Section on Maternal Request on Peripartum Anxiety and Depression in Women with Childbirth Fear: A Systematic Review. BMC Pregnancy Childbirth.

[25] Sobande A., Eskandar M. (2006). Multiple Repeat Caesarean Sections: Complications and Outcomes. J. Obstet. Gynaecol. Can..

[26] Yaman Tunc S., Agacayak E., Sak S., Basaranoglu S., Goruk N.Y., Turgut A., Tay H., Elci E., Gul T. (2016). Multiple Repeat Caesarean Deliveries: Do They Increase Maternal and Neonatal Morbidity?. J. Matern. Fetal Neonatal Med..

[27] Uyanikoglu H., Karahan M.A., Turp A.B., Agar M., Tasduzen M.E., Sak S., Erdal Sak M. (2016). Are Multiple Repeated Cesarean Sections Really as Safe?. J. Matern. Fetal Neonatal Med..

[28] Biler A., Ekin A., Ozcan A., Inan A.H., Vural T., Toz E. (2017). Is It Safe to Have Multiple Repeat Cesarean Sections? A High Volume Tertiary Care Center Experience. Pak. J. Med. Sci..

